# Emergence of Zika virus infection in China

**DOI:** 10.1371/journal.pntd.0008300

**Published:** 2020-05-19

**Authors:** Chuan-min Zhou, Jian-wei Liu, Rui Qi, Li-zhu Fang, Xiang-rong Qin, Hui-ju Han, Rong-can Mo, Hao Yu, Yong-jun Jiao, Jian-yan Lin, Xue-jie Yu

**Affiliations:** 1 State Key Laboratory of Virology, School of Health Sciences, Wuhan University, Wuhan, P.R. China; 2 Nanning Blood Center, Nanning, P.R. China; 3 Institute of Pathogenic Microbiology, Jiangsu Provincial Center for Disease Prevention and Control, Nanjing, China; 4 The Fourth People's Hospital of Nanning, Nanning, P.R. China; Faculty of Science, Ain Shams University (ASU), EGYPT

## Abstract

Currently, Zika virus (ZIKV) is spreading across the world and no ZIKV infection cases have ever been reported in China. Here, we aimed to determine whether ZIKV infection exists in China. Blood samples of 273 healthy individuals were collected from Nanning City, Guangxi Province, China in March 2019. We found that 9.5% (26/273) and 1.8% (5/273) of healthy persons were positive to ZIKV total antibody (IgG and/or IgM) IgM antibody, respectively. All ZIKV positive plasma samples were negative to Dengue virus and West Nile virus. Among the ZIKV antibody positive plasma samples, 65.4% (17/26) exhibited neutralizing activity to ZIKV. Followed up studies showed that none had clinical symptoms of ZIKV infection and oversea experience. Together, our study indicates that endemic ZIKV infections emerge in China, which not only suggested that ZIKV posed a potential threat to public health in China, but also expand the ZIKV epidemic areas in East and Southeast Asia.

## Author summary

ZIKA, caused by mosquito-borne Zika virus (ZIKV), is typically asymptomatic or with mild fever, and the occurrence of ZIKV infections are reported around the world. However, limited ZIKV epidemiological investigation was performed and no ZIKV infection cases were ever reported in China. Here, we observed that Zika virus also distributes in China. This work not only suggests ZIKV as a potential threat to public health in China but also expands our knowledge of ZIKV epidemic regions in East and Southeast Asia. Therefore, government officials, physicians and public health workers in China need to pay attention to ZIKV infection cases.

## Introduction

Zika virus (ZIKV) is a positive single-stranded RNA virus, belonging to mosquito-borne *Flavivirus*, which was first discovered in Uganda in 1947 from a sentinel febrile rhesus monkey[1,2]. ZIKV strains are now classified into African, Asian or Yap lineages based on genome sequences [3]. Adults infected with ZIKV are mostly asymptomatic or exhibited mild fever, but for newborn infants the infection caused severe microcephaly [2,3]. It should be mentioned that ZIKV remained neglected until the outbreak of ZIKV infections in Micronesia in 2007 [4]. To date, ZIKV is spreading around the world and 89 countries have reported ZIKV cases [5]. In South and Southeast Asia, 12 countries have reported ZIKV infections including Bangladesh, Cambodia, India, Indonesia, Laos, Malaysia, Maldives, Myanmar, Philippines, Singapore, Thailand, and Vietnam [5].

It has been confirmed that *Aedes aegypti*, *A*. *albopictus*, *A*. *vittatus*, and *A*. *luteocephalus*, *Mansonia uniformis*, *Culex perfuscus* and *Anopheles coustani* served as ZIKV transmitting vectors [6–12]. In China *A*. *aegypti* are found mainly distributed in the south, including Hainan, Guangdong, Guangxi, and Yunnan provinces, while *A*. *albopictus* are more extensively distributed than *A*. *aegypti* [13]. Recent laboratory study also identified that *A*. *aegypti* and *A*. *albopictus* mosquitoes in China were capable of mediating ZIKV transmission whereas *C*. *quinquefasciatus* was incapable [6]. However, another study showed contradictory results reporting that ZIKV (GZDJ1685) was isolated from *C*. *quinquefasciatus* mosquitoes in Southern China [14].

About 2.6 billion people live in areas suitable for ZIKV transmission including the Asia-Pacific region and Africa ^15^. In view of recent advances of the ZIKV distribution model, Southeast China was proven to be highly suitable for ZIKV transmission due to widespread distribution of *Aedes* and suitable environmental conditions [15]. There are more than 210 million people living in ZIKV suitable areas in China [15,16]. Additionally, a large volume of travelers floated between different ZIKV-affected areas and China each year [16]. Hence, China is becoming increasingly susceptible and vulnerable to ZIKV.

To date, no study has reported the existence of human infection with ZIKV, except for a number of imported ZIKV infection cases in China [17]. Therefore, in this study, we conducted a survey to determine the existence of ZIKV in China.

## Methods

### Sample collection and ethics statement

A total of 273 blood samples were collected from 273 healthy or asymptomatic individuals in the suburb of Nanning City (22.8170° N, 108.3665° E), Guangxi Province, China, in March 2019. All individuals were native of Nanning City without oversea experience with age distribution from 20 to 57 and gender ratio (male:female) 1.37:1. The study was reviewed and approved by the ethics committees of Wuhan University. Written informed consent was obtained from each person. Plasma samples were collected using 5ml BD Vacutainer Barricor plasma blood collection tubes. Plasma was immediately stored at –80°C.

### Nested-PCR

Total RNA was extracted from plasma samples using the RNeasy Mini Kit (QIAGEN, Hilden, Germany). cDNA was synthesized using Reverse Transcription System (Promega, Madison, WI, USA). Nested-PCR first amplification primers (5’-CCATCTGGTACATGTGG-3’ and 5’-CATGTCCTCAGTRGTCATCC-3’); second amplification primers (5’-GTGGAGATGACTGCGTTGTGAAGCC-3’ and 5’-CCA TCAGTCGAAGGTCTCTTCTGTGG-3’) [18].

### ELISA

Human plasmas were measured using a ZIKV-NS1-IgM ELISA Kit (Wending Biotech, Nanjing, Jiangsu, China), total ZIKV NS1 antibody ELISA Kit (Wending Biotech, Nanjing, Jiangsu, China), Dengue virus (DENV)-IgG ELISA Kit (Zhongshan Biotech, Zhongshan, Guangdong, China) or West Nile Virus (WNS) IgG DxSelect (FOCUS Diagnostics, CA). Briefly, to detect the ZIKV-NS1-IgM or ZIKV NS1 total antibody, ZIKV NS1 or anti-human IgM monoclonal antibody pre-coated polystyrene plates were incubated with 50 μl sample dilution buffer combined with 50 μl negative control, positive control, or plasma samples. After 30min incubation at 37°C, plates were washed with wash buffer for 5 times. HRP-conjugated-ZIKV NS1 antigen (100 μl) was coated with plates for 30min at 37°C followed by 5 times wash with wash buffer. In the end, plates were incubated with 3,3',5,5'-tetramethylbenzidine substrate for 5min and the reaction was stopped with stop solution. Optical density (OD) was read at 450 nm in an absorbance microplate reader. The mean value OD of negative control is less than 0.1. Cut off = 0.748* mean value OD of negative control+0.146.

### Virus isolation

Plasma samples were centrifuged at 13,523 g for 10min with a microcentrifuge and sterilized using 0.45 μm microfilters. The plasma samples were then diluted by a factor of 10 and inoculated with Vero cells. Vero cells were incubated at 37°C in 5% carbon dioxide and observed daily for cytopathic effects (CPE). At the same time, RNA was also isolated to detect ZIKV using nested-PCR.

### Micro-neutralization assays

Micro-neutralization assays were then used to detect neutralizing antibodies against ZIKV. ZIKV was kindly provided by Jiangsu Provincial Center for Disease Prevention and Control [19]. Briefly, the negative plasma control and ZIKV positive plasma samples were pre-treated at 56°C for 30min. Plasma samples were diluted serially from 1:2 to 1:64 and mixed with an equal volume of 100 TCID_50_ of ZIKV. The mixture was incubated at 37°C for 2 hours and then added into Vero cells in triplicate in 96-well tissue culture microplates. Vero cells were checked for 10 days to evaluate CPE in 2% FBS DMEM medium. Crystalline Violet staining was then used to monitor the CPE of different dilution ratios [20].

## Results

**Fig 1:**
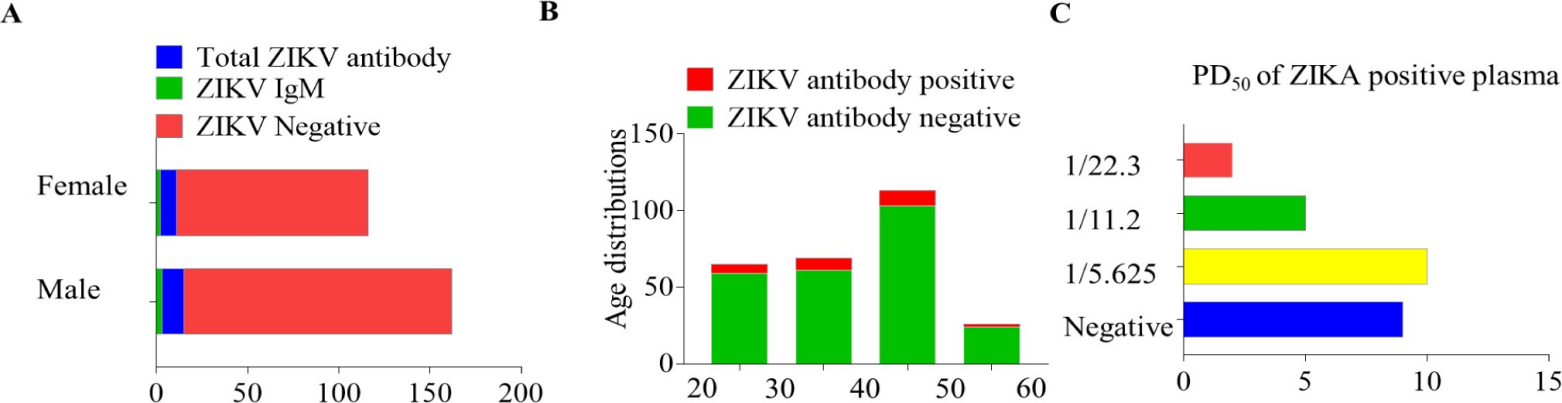
Demographic characteristics of 273 blood donators and ZIKV positive individuals. (A) ZIKV antibody histograms of 273 blood donators; (B) age distributions histograms of 273 blood donators; (C) Micro-neutralization assay results of ZIKV positive plasma. PD_50_ (50% protective dose).

**Fig 2:**
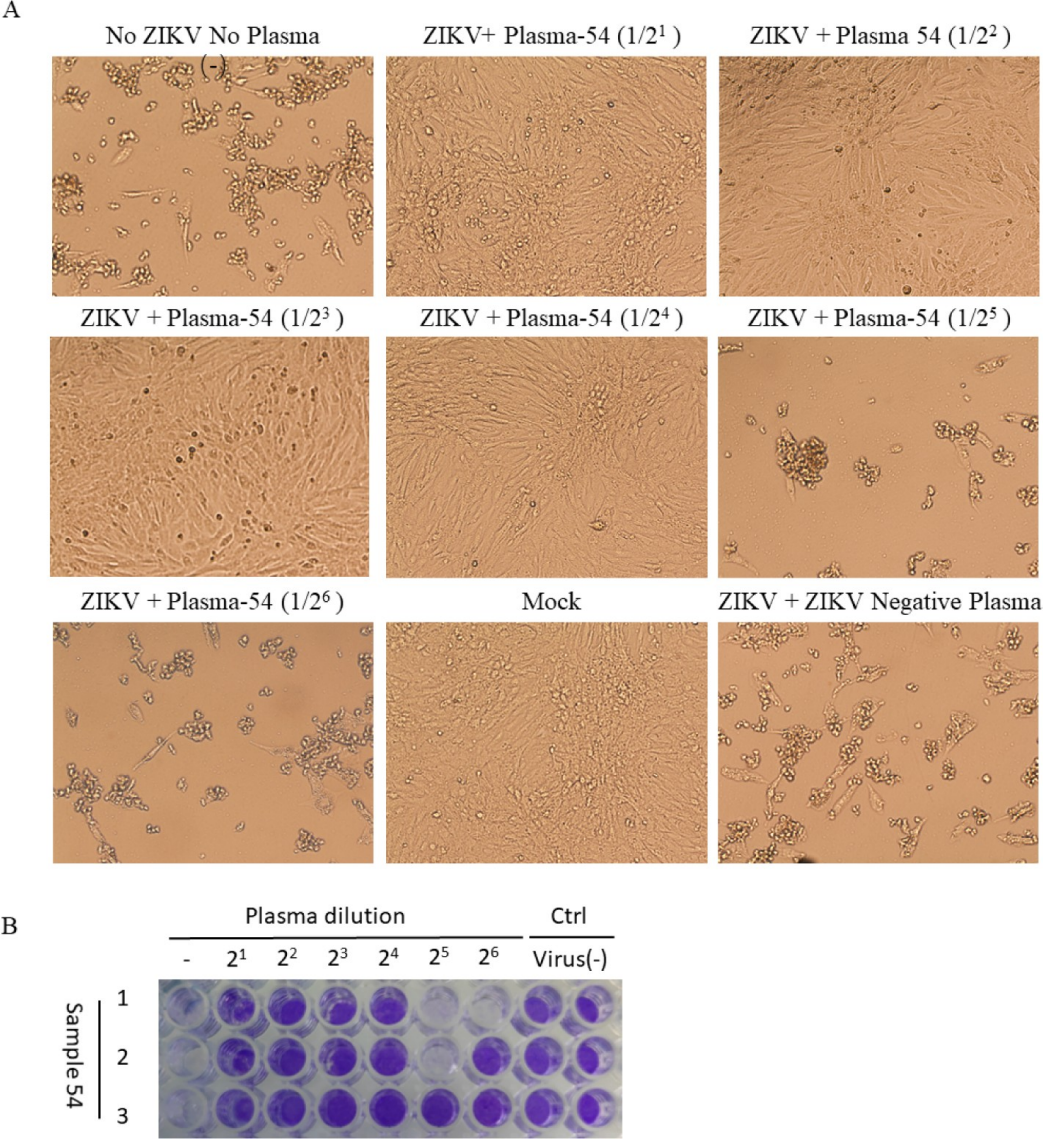
(A, B) Micro-neutralization assays of plasma sample-54 (representative assay) at different dilution with ZIKV in Vero cells. Vero cells were checked for 10 days to monitor CPE.

A total of 273 healthy individuals were surveyed in the suburb of Nanning City, Guangxi Province, China. All plasma samples were analyzed for ZIKV antibodies with antigen sandwich-ELISA. ELISA results showed that 9.5% (26 /273) of healthy human plasma samples were positive to total ZIKV antibodies (IgG and IgM) and 1.8% (5/273) of healthy individuals were ZIKV IgM positive **(Fig 1A; Table 1)**. In addition, ELISA results showed that all 26 ZIKV positive plasma samples were negative to DENV and WNV. ZIKV RNA fragments were not detected by nested-PCR and ZIKV was not isolated in the 5 ZIKV IgM positive plasma samples **(Fig 1A)**. Among those ZIKV positive individuals, 15 were male and 11 were female, with a sex ratio of 1.36. Most of them were between 30 and 50 years of age **(Fig 1B)**. Micro-neutralization assays were then used to detect the neutralization antibodies in ZIKV positive plasma samples. We found that 17 ZIKV antibody positive plasma samples showed neutralizing activity up to 1/22 **(Fig 1C and Fig 2)**. Twenty-six individuals with ZIKV antibody were then followed up one by one for 2 months after they were tested positive, and they all lacked clinical symptoms of ZIKV infection. In addition, none of them had oversea experience.

**Table 1:** Characteristics of 273 blood donators and ZIKV positive individuals.

	Age (years)	20~29	30~39	40~49	50~59
ZIKV positive	Male (cases)	3	4	7	1
	Female (cases)	3	4	3	1
ZIKV negative	Male (cases)	29	33	59	23
	Female (cases)	30	28	44	1

## Discussion

*Flavivirus* genus, including DENV, yellow fever virus, Japanese encephalitis virus, and WNV, have caused countless infections for decades and posed a serious threat to public health in the world [21]. ZIKV also belongs to the *Flavivirus* genus. Discovery of ZIKV was first noticed in Uganda in 1947, but only sporadic ZIKV infections were reported until its outbreak in Micronesia [1,2]. To date, ZIKV is spreading globally and the occurrence of ZIKV infection is reported around the world, which raises global concern. Despite observing ZIKV infections in 12 South and Southeast Asian countries, current knowledge about ZIKV especially in East Asia and China, is still limited.

Previous studies have reported isolation of ZIKV from mosquitoes and travelers in Guizhou Province, China [6,14]. However, the existence of endemic ZIKV infection in China was not reported up to date. In this study, we demonstrated that healthy individuals were serological positive to ZIKV NS1 IgG and/or IgM by ELISA. We also demonstrated that 17 plasma samples of the 26 persons have micro-neutralization (MNT) antibodies [22]. Considering the cross-reaction may exists among flaviviruses, detection of DENV and WNV was analyzed by ELISA and the results was negative in all 26 ZIKV positive plasma samples. Additionally, previous report has showed that NS1 of flaviviruses is highly specific and there is no cross-reaction between ZIKV-NS1 and Japanese encephalitis virus (JEV) NS1 proteins [23,24]. We have also analyzed the sequence homology of NS1 proteins among flaviviruses and we found that the amino acid sequence of the NS1 proteins similarity was very low among flaviviruses. The sequence homology between ZIKV and JEV, WNV or DENV was 57%, 56% and 54%, respectively. The conserved sequences of ZIKV and JEV are consistent with the conserved sequences of ZIKV and WNV or DENV. Therefore if the ZIKV positive sera react with JEV, it should also react with WNV and DNEV. Hence, our data suggest that the ZIKV reaction antibodies in human plasma were induced by ZIKV infections. We were unable to detect ZIKV RNA or isolate ZIKV from IgM positive healthy individuals. We hypothesized that the viral load might be very low or viremia period is very short in these people.

Most ZIKV infections were asymptomatic or caused mild and self-limiting symptoms, such as fever and headache [25]. We noticed that all ZIKV positive individuals showed no clinical symptoms of ZIKV infection and no obvious discomfort before and after blood collection. Remarkably, ZIKV is capable of causing severe birth defects through vertical transmission. Hence, considering the high asymptomatic infection rate found in Nanning City, our study reminded us that the risk for potential ZIKV outbreaks cannot be ignored in China.

**Fig 3:**
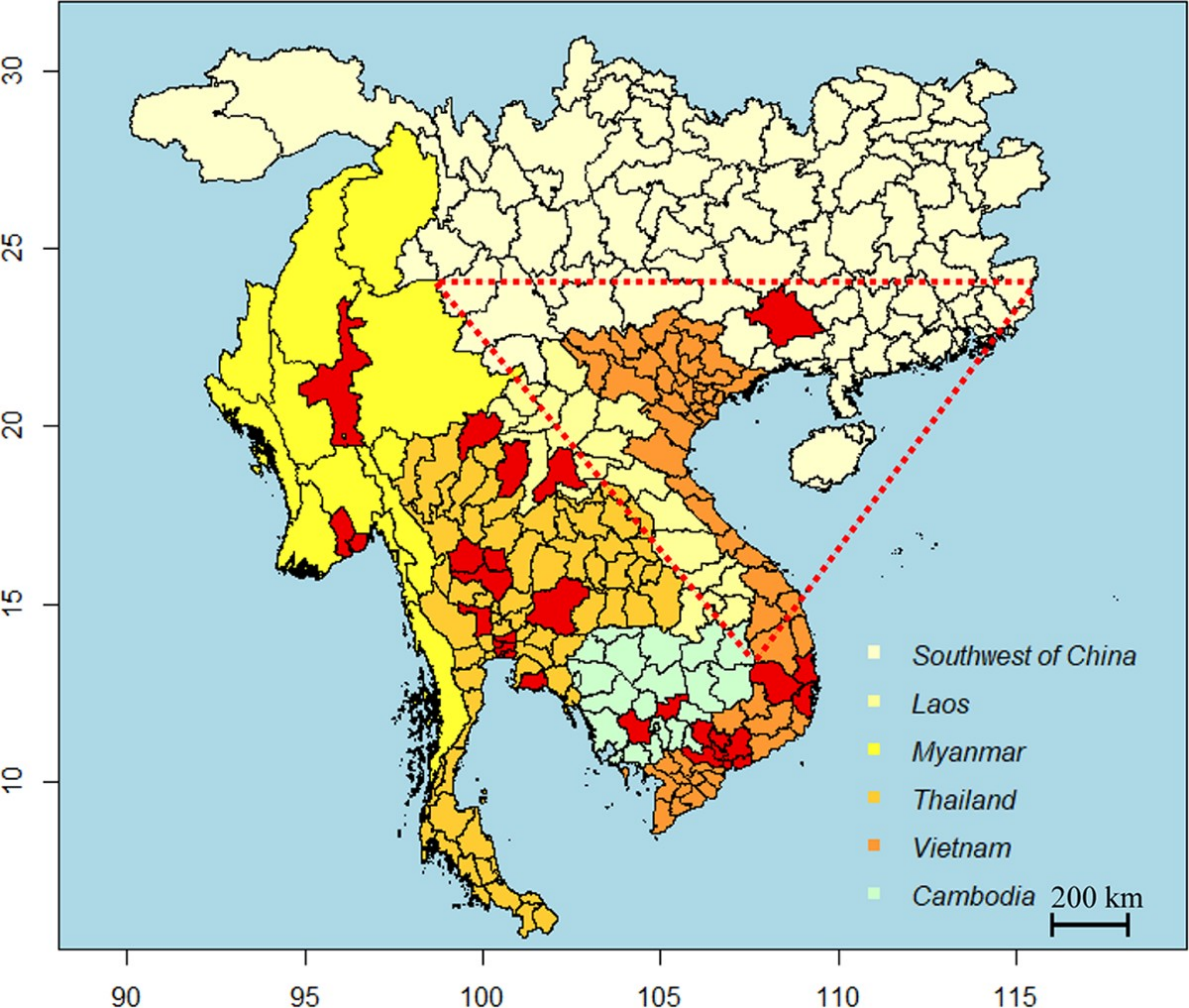
ZIKV occurrence location records in China and part of Southeast Asian countries including Vietnam, Laos, Myanmar, Cambodia and Thailand; Red areas indicated ZIKV occurrence locations; The dotted red line indicated ZIKV high risk area.

All plasma samples from healthy individuals were collected in the suburb of Nanning City, which is located in Southeast China. Our followed-up study showed that all ZIKA positive individuals are native to Nanning City and none of them had oversea experience, indicating that they're massively more likely that the ZIKV detected in China is endemic. Additionally, Nanning City is located in the south of Guizhou Province [14], where ZIKV was isolated from mosquitoes. Hence, it is not surprising that ZIKV existed in Nanning City. We then analyzed the records of locations of ZIKV occurrences in parts of Southeast Asian countries. We noticed that no ZIKA infection cases were ever reported in territories of Southeast Asian countries that are located up to 800 km to the south and west of Nanning. In other words, the ZIKV infection cases in Vietnam, Laos, and Thailand mainly concentrated on these countries’ southern regions and no ZIKV cases were ever reported in their northern regions. **(Fig 3)**. It is worth mentioning that the occurrence of ZIKV infections were mostly distributed in the south and the central Highlands in Vietnam [26].

In view of that Nanning City located close to to Vietnam and Guizhou, and share similar environmental conditions and mosquito distributions, our research revealed and supported that ZIKV infections existed in Nanning City, which strongly suggested that ZIKV epidemic areas have already extended to most of the areas between the northern part of Southeast Asian countries and Nanning City **(Fig 3)**. Hence, our study not only reported that distribution of ZIKV in China is endemic but also broadened the area of ZIKV epidemic regions and supported the ZIKV distribution model in East and Southeast Asia [15,16].

In all, we demonstrated endemic ZIKV infections in China. Our study expanded our knowledge of ZIKV epidemic regions in East and Southeast Asia. From our report in this study and sporadic reports of ZIKV infections in humans in Southeast Asian countries including Southern Vietnam, Western Laos, Southern Thailand, Cambodia, and Southern Myanmar, we deduced that ZIKV infection in humans may distribute broadly from Guangxi Province, China to Southeast Asian countries. Further study is also necessary to detect whether other parts of China, especially in Southern China, exhibit ZIKV positive. People living in these areas are at high risks of ZIKV infection. This information will alert government officials, physicians and public health workers in China and Southeastern Asian countries to pay attention to ZIKV infection cases; and physicians around the world to pay attention to ZIKV infections for travelers who have returned from Southern China and Southeast Asia.
